# Evaluation of the Stability of Concentrated Emulsions for Lemon Beverages Using Sequential Experimental Designs

**DOI:** 10.1371/journal.pone.0118690

**Published:** 2015-03-20

**Authors:** Teresa Cristina Abreu Almeida, Ariane Leites Larentis, Helen Conceição Ferraz

**Affiliations:** 1 Chemical Engineering Program, COPPE, Universidade Federal do Rio de Janeiro, Rio de Janeiro, RJ, 21941-972, Brazil; 2 Centro de Estudos da Saúde do Trabalhador e Ecologia Humana (CESTEH), Escola Nacional de Saúde Pública (ENSP), Fiocruz, Rio de Janeiro, RJ, 21041-210, Brazil; The University of Tokyo, JAPAN

## Abstract

The study of the stability of concentrated oil-in-water emulsions is imperative to provide a scientific approach for an important problem in the beverage industry, contributing to abolish the empiricism still present nowadays. The use of these emulsions would directly imply a reduction of transportation costs between production and the sales points, where dilution takes place. The goal of this research was to evaluate the influence of the main components of a lemon emulsion on its stability, aiming to maximize the concentration of oil in the beverage and to correlate its physicochemical characteristics to product stability, allowing an increase of shelf life of the final product. For this purpose, analyses of surface and interface tension, electrokinetic potential, particle size and rheological properties of the emulsions were conducted. A 2^4-1^ fractional factorial design was performed with the following variables: lemon oil/water ratio (30% to 50%), starch and Arabic gum concentrations (0% to 30%) and dioctyl sodium sulfosuccinate (0 mg/L to 100 mg/L), including an evaluation of the responses at the central conditions of each variable. Sequentially, a full design was prepared to evaluate the two most influential variables obtained in the first plan, in which concentration of starch and gum ranged from 0% to 20%, while concentration of lemon oil/water ratio was fixed at 50%, without dioctyl sodium sulfosuccinate. Concentrated emulsions with stability superior to 15 days were obtained with either starch or Arabic gum and 50% lemon oil. The most stable formulations presented viscosity over 100 cP and ratio between the surface tension of the emulsion and the mucilage of over 1. These two answers were selected, since they better represent the behavior of emulsions in terms of stability and could be used as tools for an initial selection of the most promising formulations.

## Introduction

The beverages market requires constant releases of innovative products, aiming to follow up on demands of consumers and trends such as the requirement of soft drinks with specific nutrients or low calorie counts, besides ensuring the company's competitiveness. All these factors generate the need for agility in the formulation and development of new products.

Most beverages are formed by oil-in-water (o/w) emulsions, which must be stable in concentrated and in diluted forms, as well as in carbonated form, in the case of soft drinks. One of the most common indicators of deterioration of an emulsion in a soft drink is the formation of an opaque/oily ring around the neck of the bottle. In general, the base emulsion for forming the drink is produced in the industry and transported to the bottling and sale posts, where it is diluted and carbonated to obtain the final product. Among the main factors that affect the quality of the end product, stability of the base emulsion is certainly the most important.

Emulsions for foodstuffs can become unstable due to several physicochemical mechanisms [1, 2, 3] such as flocculation, flotation, sedimentation, coalescence, Ostwald ripening and phase inversion. It bears noticing that these destabilizing mechanisms are normally correlated; for instance, there is an increase in particle size due to aggregation by flocculation, coalescence or Ostwald ripening. This normally results in an increase in instability of the drops and thus leads to gravitational separation (flotation/sedimentation). Additionally, these processes may happen simultaneously, not only consecutively [4, 5, 6].

The identification and, especially, the comprehension and control of factors that affect the stability of emulsions have been object of intensive studies since the 1980s. The fraction of oil, the type and concentration of surfactant and stabilizing agents and the difference of density between phases are some of the variables that exert the highest influence on the shelf life of an emulsion. In literature, there are several studies about the effect of these variables on the stability of emulsions for beverages [5, 7, 8, 9, 10]. The vast majority of emulsions for beverages studied have an oil concentration of up to 20% or emulsions for cosmetics with an oil concentration of approximately 80%. The experimental design tool is used by many authors, aiming to determine the best conditions to obtain more stable emulsions.

Thus, the most important goals in this field are to monitor the quality of emulsions during and after production, developing new formulations, ensuring that the properties previously stipulated are matched and predicting how the final product will behave during storage and at the sales point [4]. However, due to the complexity of its nature, the formulation of a specific emulsion microstructure and prediction of its final properties are very challenging tasks [10]. There are few studies in literature related to concentrated emulsions for beverages, with oil content over 50%. The use of these emulsions by the beverage industry would be of great interest, as it would directly imply a reduction of transportation costs between the point of production and the sales point, where dilution takes place.

In this context, the aim of this work was to investigate the main factors affecting stability of concentrated emulsions for beverages employing experimental design techniques based on variables that are easy to assay, allowing the selection of formulations capable of producing beverages with increased shelf life. From the variables evaluated, we sought to identify which responses could better translate the initial stability of the emulsion to prediction of stability of concentrated lemon oil emulsions.

## Materials and Methods

As oil phase, we employed essential lemon oil (Mapric), broadly used in soft drinks in Brazil and for which there are few studies in the literature. As stabilizing agents, we selected Arabic gum (Vetec), a natural resin frequently used as thickener and stabilizer for several foods, due to its low cost and efficiency as a stabilizing agent in emulsions [11]. We also investigated the use of Purity Gum 2000 modified starch (kindly donated by National Starch Food Innovation), a corn byproduct recommended for replacing Arabic gum as a stabilizing agent in emulsions for food products. As a surfactant, we used DSS (Dioctyl sodium sulfosuccinate, Sigma-Aldrich), an anionic surfactant that is highly used in the non-alcoholic carbonated beverage industry. We also added to the emulsions citric acid (Vetec), sodium benzoate (Vetec) and potassium sorbate (Vetec) in fixed amounts, as conservatives, antioxidants and bactericidal agent, respectively. Finally, sucrose hexaisobutyrate diacetate (SAIB, Sigma-Aldrich) was added to the oil phase, so as to equalize the density of the oil and water phases, minimizing separation due to gravitational action (flotation or sedimentation) [12].

### Sequential design of experiments

Initially, a 2^4-1^ fractional factorial experimental design was implemented, comprised of eight (8) experiments + three (3) central points (Table 1). The following parameters were varied in this experimental matrix: the volumetric fraction of the oil phase in the emulsion (30% to 50% in volume), the ratio of modified starch/water and Arabic gum/water (from 0% to 30%, grams of Arabic gum or starch mass in 100 grams of water) and the concentration of DSS surfactant (from 0 mg/L to 100 mg/L of DSS in water), as allowed in Brazilian legislation. Experiments in the central point were conducted on different days. Global experimental error includes influence of equipment errors, day of the experiment, temperature variation, the experimenter’s hand, stabilization of the electric network, among others.

**Table 1:** Range of the independent variables values in the sequential experimental design strategy.

	Variable	Code	-1	0	+1
Firstdesign	Oil	-	30% o/w	40% o/w	50% o/w
	Starch	-	0% g/g H_2_O	15% g/g H_2_O	30% g/g H_2_O
	Arabic gum	-	0% g/g H_2_O	15% g/g H_2_O	30% g/g H_2_O
	DSS	-	0 ppm	50 ppm	100 ppm
Second design	Starch	x_1_	0% g/g H_2_O	10% g/g H_2_O	20% g/g H_2_O
	Arabic gum	x_2_	0% g/g H_2_O	10% g/g H_2_O	20% g/g H_2_O

As indicated in Table 1, after conclusion of the fractional factorial experimental design (first design), a two-level full-factorial experimental design was implemented (second design), varying the two statistically significant variables obtained in the first evaluation. Since two variables were chosen, the design consisted of four (4) experiments + three (3) central points, as per Table 1. In the second experimental design, the modified starch/water and Arabic gum/water ratio was adjusted to a new range, from 0 to 20%.

Statistical analyses were performed using *Statistica 8*.*0* (StatSoft) software, for analysis of the effects of each variable and their interactions. For the fractional factorial experimental design, due to its exploratory nature, analyses were performed employing 90% of confidence, avoiding the exclusion of variables that, in a more conservative statistical analysis, would not present a significant effect [13, 14]. Analyses were performed employing a higher confidence level of 95% for the full-factorial experimental design. However, visual analyses (amount of oil released and stability after 15 days) were evaluated with a 90% confidence level (*p* ≤ 0.10), so as not to exclude possible relevant factors/variables that would not be included in an analysis with a 95% significance level, since the inherent errors of these measurements are high.

The significance of each linear effect and interactions were determined in the full-factorial experimental design using the t test at a probability level of 0.05 (95% confidence level). The *p*-value represents the probability of a given variable having a non-significant effect on the response, that is, it has a 5% probability of not being significant. Effects were statistically significant when the *p*-value was less than 0.05. The effects estimated indicated the strength of the main effects and their interactions.

### Emulsion preparation

All aqueous phases, herein called mucilage, had fixed concentrations of the following additives: 1.6% (w/w) citric acid, 0.4% (w/w) sodium benzoate and 0.4% (w/w) potassium sorbate. All additives were water-soluble at room temperature. The amount of starch and Arabic gum varied according to the experimental design employed (Table 1). Water was slowly added to previously weighted materials. Afterwards, a high-speed homogenizer (*Ultra-Turrax T 25 digital*, *IKA*) was used to promote mixing, 4,000 rpm for 5 minutes. Finally, the mucilage was left to rest for 24 hours to get rid of any formed foam.

The oil blend was prepared from Sicilian lemon oil with weighting agent SAIB so that the densities of the water and oil phases were as close as possible.

The emulsions were produced using a high-speed homogenizer, adding the oil little by little into the mucilage, which was already stirring at 4,000 rpm with Turrax. After all of the oil was poured in, the system was stirred for 15 minutes at 8,000 rpm.

### Characterizations

The characterizations were performed only at instant zero, right after the emulsions were produced, to determine their initial stability. An exception was the de-emulsification index analysis, which was monitored during 15 days after production of the emulsion.

#### Surface/interfacial tension

All measurements were performed through the Du Nouy ring method, using a recipient of 40 mL, with the *Sigma 70 tensiometer* (KSV Instruments Ltd). Surface tensions of the mucilages, the oil phase containing SAIB and emulsions were determined. In addition, interfacial tension between the mucilage and the oil without SAIB was evaluated.

#### De-emulsification Index

This is a very simple analysis, conducted only through visual observation, consisting of measuring the height of the oil layer (H_O_) in relation to the total height of the emulsion (H_E_) [4]. Stability after 15 days was measured by calculating the maximum layer of oil (H_TO_) that could be formed when complete separation takes place (H_TO_ = 0.3H_E_, 0.4H_E_ or 0.5H_E_, depending on the oil content), as per Equation 1. The height reached by the emulsion was recorded and, at the fifteenth day, the height reached by the released oil was measured. Pictures were taken at both moments to show the change in emulsion stability. Emulsion stability index was then calculated from Equation 2.

$$\mathrm{De}-\mathrm{emulsification index}=100\times \frac{H_{O}}{H_{E}}$$ (1)

$$\mathrm{Stability}\;\left(15\;\mathrm{days}\right)=100\;\times \;\left(1-\frac{H_{O}}{H_{TO}}\right)$$ (2)

#### Particle Size

Particle size distribution was characterized with the *Dynamic Light Scattering Particle Size Analyzer LB550* (Horiba). Average droplet size was characterized in terms of the volume mean diameter *d*_4,3_, obtained from histogram, defined in Equation 3:

$$d_{4,3}=\frac{\sum_{i}n_{i}\;{\text{d}}_{i}^{4}}{\sum_{i}n_{i}\;{\text{d}}_{i}^{3}}$$ (3)

where *n*_*i*_ is the number of the droplets of diameter *d*_*i*_. For this measurement, samples of the just-prepared emulsions were previously diluted 1000x, in the first fractional experimental design, and 500x, in the second full-factorial design. Ultra-pure water was used for the dilution. The refraction index assumed for the oil was 1.468, as previously determined.

#### Zeta Potential (ζ)

ζ measurement was performed with *ZetaPlus* (Brookhaven Instruments Corporation). For this measurement, samples of just-prepared emulsions were previously diluted with ultra-pure water, 1000x in the first fractional factorial design, and 500x in the second full-factorial experimental design.

#### Rheometry

The following rheometers were used to analyze the rheological properties of emulsions: *Haake Rheo-Stress 1* (Thermo Fisher Scientific) and *Advanced Rheometer AR 2000* (TA Instruments), for the first fractional design, and *AR G2* (TA Instruments) for the second full-factorial design. The analysis was performed in continuous shearing to determine viscosity of the emulsions, mucilages and oil blend. The shear rate varied from 0.01 to 1000 1/s and back from 1000 to 0.01 1/s, at a time interval of 300 s, while measuring the shearing tension at each point, allowing determination of the viscosity. At the first design, a cone and plate titanium assembly with 60 mm and 1° was used. In the second design, the assembly was similar, the angle being 2°.

## Results and Discussion

The experimental design technique has received increasing attention in the emulsion stability field in recent years [5, 7, 8, 9, 10]. The goal is to obtain quality information about factors/variables that affect the properties of emulsions, especially their stability. Additionally, it is possible to acquire a better comprehension of the system and to improve the preparation process. The possibility of predicting stability before producing certain emulsion formulations would very appealing to the industry. In this scenario, emulsion stability would be determined by its microstructure, monitored through its physicochemical properties.

The experimental strategy employed in this work was to perform a sequential design of experiments, beginning with a fractional factorial experimental design for selection of the significant variables involved in the production of concentrated o/w emulsion for beverages. Afterwards, a full-factorial experimental design was implemented to evaluate effects and interactions of statistically significant variables in the first design. It was thus possible to evaluate the effects of the variables, to obtain linear models for some properties of the emulsion and to determine optimal conditions of stability for the system being studied.

The responses analyzed were the difference between density of mucilages and density of oil phase with SAIB (Δρ), the ratio of emulsion surface tension and mucilage surface tension (γ emulsion/ γ mucilage), droplets ζ, particle size, viscosity of the emulsions measured at a shear rate of 1000 1/s, the ratio of viscosity of the mucilages and viscosity of the oil blend (η mucilage/ η oil), amount of oil released (% oil released) and stability after 15 days of production of the emulsions.

Densities of the mucilages and of the oil were measured with the goal of evaluating whether the difference between them was small enough, as a way to reduce the probability of there being a separation due to the action of gravity, flotation or sedimentation [5, 15]. Surface/interfacial tensions of the emulsions, mucilages and oil were measured with the intent of evaluating the intermolecular interactions and as an indirect measure of the free energy of the system, which directly influences the system’s stability [15, 16].

Measurement of ζ of the oil droplets in the emulsions is an indication of the role of electrostatic forces on stability of the emulsion. In order for an emulsion to be considered stabilized only by electrostatic repulsion, the ζ must be high enough to overcome attractive van der Waals interactions. There is no consensus regarding minimum ζ value required to confer electrostatic stabilization to an emulsion and it varies from one system to another, but references can be found in literature to |25|mV and to |30|mV [9].

The average particles size and the size distribution curve provide an indication of the possible destabilizing mechanisms for the emulsion. Large drops tend to suffer flotation/sedimentation. Drops too small increase the total interfacial area of the emulsion, which implies a high Gibbs free energy, leading to coalescence. Finally, a broad size distribution leads to the occurrence of Ostwald ripening [4].

The study of the rheological characteristics of emulsions, mucilages and oil shows how these materials deform and flow under the influence of external forces. The rheological properties of a given material are determined by factors that are intrinsic and circumstantial in nature, that is, they depend on the properties of the material itself (what it is made of) and on the circumstances/conditions to which it is submitted (temperature, pressure, intensity of the applied force, among others). Viscosity describes the friction between the internal layers of the fluid, which creates a resistance to the flow, as a function of the shearing, which is the necessary force to cause the movement of layers.

Finally, the amount of oil released and the stability of each emulsion were analyzed after 15 days.

### Fractional factorial experimental design

The first stage of the sequential experimental design strategy consisted in performing the experiments of the fractional factorial experimental design. The following variables were evaluated with the aim of identifying those most significant: density difference, ratio of emulsion to mucilage surface tension, droplets ζ, particle size, viscosity at a shear rate of 1000 1/s, ratio of mucilage to oil viscosity, amount of released oil and stability after 15 days. The two-level fractional factorial design is presented in Table 2.

**Table 2:** Responses obtained in the 2^4-1^ fractional factorial experimental design and triplicate at the central points to calculate the average, standard deviation and relative standard deviation.

Emulsion	Oil	Starch	ArabicGum	DSS	Δρ (muc-oil)(g/cm^3^)	γ emulsion(mN/m)	γ mucilage(mN/m)	γ emulsion/γ mucilage	ζ(mV)	Particle size(nm)	Viscosity at γ^.^ = 1000 1/s (cP)	η mucilage/ η oil	Oil released(%)	Stability after 15 days(%)
1	-1	-1	-1	-1	0.01	28.0	50.0	0.56	-34	7	4	0.1	30	0
2	+1	-1	-1	+1	0.00	27.9	33.9	0.82	-45	2468	5	0.1	50	0
3	-1	+1	-1	+1	0.06	40.9	30.0	1.36	-15	2003	107	4.2	0	100
4	+1	+1	-1	-1	0.07	41.7	30.8	1.35	-15	5539	287	5.1	0	100
5	-1	-1	+1	+1	0.08	44.4	35.3	1.26	-29	2632	163	5.4	0	100
6	+1	-1	+1	-1	0.07	48.7	43.7	1.11	-27	2543	289	5.4	0	100
7	-1	+1	+1	-1	0.15	48.7	32.0	1.52	-26	2512	401	65.2	5	85
8	+1	+1	+1	+1	0.12	82.9	32.1	2.57	-28	2618	895	30.2	0	100
9	0	0	0	0	0.07	43.5	30.5	1.43	-14	2180	160	2.6	18	55
10	0	0	0	0	0.07	44.5	32.3	1.38	-19	2866	110	3.1	17	58
11	0	0	0	0	0.10	43.6	32.5	1.34	-14	2233	169	7.2	19	52
Average at the central points					0.08	43.9	31.8	1.38	-16	2426	146	4.3	18	55
Standard deviation at the central points					0.02	0.6	1.1	0.05	3	382	32	2.5	1	3
Relative standard deviation at the central points (%)					22	1.3	3.5	3.3	18	16	22	59	6	5

Due to the addition of the weighting agent to the oil phase, its density became close to that of the water phase (Table 2) as required to reduce the gravitational separation by flotation or sedimentation [5, 15].

Surface tensions of the emulsions and mucilages were compared (Table 2), and it was possible to observe that unstable emulsions presented a surface tension lower than the respective mucilages. An explanation for this observation is that any minimal oil release from a less stable emulsion, although imperceptible to the naked eye, would lead to a reduction in the emulsion surface tension, since the oil at the surface has a lower surface tension than the mucilage.

Regarding ζ, it can be observed that, although it is a useful response for characterizing the magnitude of electrostatic effects in the system, this response did not prove sufficiently sensitive to translate to emulsion stability. That is, even for a high value of ζ in emulsion 1 (which contains neither Arabic gum nor starch) the emulsion is highly unstable and separates almost instantly. The found value was in the range mentioned in the literature [9] as high enough to stabilize an emulsion by electrostatic repulsion (over |25| mV or |30| mV) however, in this study this was not indicative of stability, as can be observed through the other responses. This indicates that the main stabilizing mechanism is not electrostatic repulsion, but rather steric repulsion. In their studies, Mirhosseini et al. [9] evaluated orange emulsions for beverages and found ζ values over |25| mV. From that, they concluded that this value was high enough to avoid flocculation. In our study, emulsions 3, 4 and the central points presented a value below |25| mV, but were very stable (Table 2).

For most emulsions, the particle sizes presented values between 2 μm and 3 μm. Exceptions were emulsion 4, with 5 μm droplets, and emulsion 1, which was so unstable that no particles were found in the sample at the moment of the size measurement (Table 2). Particle also showed a narrow size distribution curve. This fact is important because small and uniform particles reduce incidence of destabilizing mechanisms such as Ostwald ripening and flotation, and also improve the flavor of the product [4].

Regarding rheological behavior of the emulsions (Table 2), it was observed that all emulsions were pseudoplastic, presenting a decrease in viscosity with the increase in shear rate. The mucilages and oil blend were characterized as Newtonian fluids, since the viscosity remains constant with the increase in shear rate. The ratio between the viscosity of mucilages and oil blend (η mucilage/ η oil) was also analyzed. Low viscosity in a continuous medium may favor collision between drops. On the other hand, high viscosity may cause an elongation of the drop, a viscous stress, which can lead to its rupture [4]. One of the central points presented a very high η mucilage/ η oil ratio; however, upon performing the statistical analysis with or without the inclusion of this point, the same variables were statistically significant considering a 90% confidence level (as will be shown further on in Table 3), indicating that, despite the high error, this point did not compromise the analysis of data of the fractional experimental design [17].

**Table 3:** Effect (± standard error) of the independent variables on the density difference between the mucilage and the oil+SAIB (Δρ), the ratio between the surface tension of the emulsion and of the mucilage (γ emulsion/ γ mucilage), the ζ, particle size, viscosity and ratio between the viscosities of the mucilage and of the oil+SAIB (η mucilage/ η oil), amount of oil released and the stability of the emulsion after 15 days, in the 2^4-1^ fractional factorial design.

Factor	Δρ (muc-oil)(g/cm^3^)	γ emulsion/γ mucilage	ζ(mV)	Particle size(nm)	Viscocity at γ^.^ = 1000 1/s (cP)	η mucilage/ η oil	Oil released(%)	Stability after15 days (%)
Mean/Interc.	**0.07 ± 0.00**	**1.34 ± 0.05**	**-24 ± 3**	**2509 ± 354**	**235 ± 43**	**11.7 ± 4.4**	**13 ± 4**	**68 ± 10**
Oil	-0.01 ± 0.01	**0.29 ± 0.13**	-3 ± 7	1504 ± 830	**200 ± 101**	-8.5 ± 10.2	4 ± 10	4 ± 23
Starch	**0.06 ± 0.01**	**0.76 ± 0.13**	13 ± 7	1256 ± 830	**307 ± 101**	**23.4 ± 10.2**	**-19 ± 10**	**46 ± 23**
Arabic gum	**0.07 ± 0.01**	**0.59 ± 0.13**	0 ± 7	72 ± 830	**336 ± 101**	**24.2 ± 10.2**	**-19 ± 10**	**46 ± 23**
DSS	-0.01 ± 0.01	**0.37 ± 0.13**	-4 ± 7	220 ± 830	47 ± 101	-9 ± 10.2	4 ± 10	4 ± 23

Statistically significant variables (*p*-value < 0.1) are highlighted in bold.

Amount of released oil and stability after 15 days (Table 2) showed a similar behavior, but opposed to one another. This relationship was expected, since the amount of oil released from an emulsion is related to the destabilization of the system.

Analyses of the effects of independent variables (oil, starch, Arabic gum, DSS) on the previously specified responses are presented in Table 3. Variables with statistically significant effects on Δρ were concentration of modified starch and of Arabic gum. Both products are commonly used as stabilizers and thickeners, increasing viscosity of the aqueous phase [16, 18]. The positive effects indicate that the increase in concentration of starch and Arabic gum results in an increase of Δρ. However, Δρ was always inferior to 0.1 g/L, for all evaluated emulsions in this experimental design, due to the use of the weighting agent (SAIB). This should be enough to avoid phase separation by mechanism of gravitational separation. The net effect of increasing starch and Arabic gum concentration is to increase stability, which can be attributed to entropic stabilizing effects, reducing the driving force towards phase separation.

As to the ratio between surface tension of the emulsion and mucilage, (γ emulsion/ γ mucilage), all variables were statistically significant (Table 3). Oil content had a positive effect on the ratio of surface tensions; this means that the increase of the oil amount did not cause a release of oil to the emulsion surface. Instead, high oil content may have lowered flotation/sedimentation speed, contributing to increase emulsion stability [5]. Modified starch and Arabic gum are stabilizing agents, increasing emulsion stability and reducing the release of oil. Finally, DSS is a surfactant, which adsorbs into the oil/water interface, decreasing interfacial tension [15, 16]. As such, emulsion formation is facilitated and stability is increased. Therefore, as expected, it was a significant variable with a positive effect on the γ emulsion/ γ mucilage ratio.

As previously discussed for results in Table 2, ζ was not related to emulsion stability. Statistical analysis corroborates this finding, since none of the variables had any effect on ζ at the studied range, although the effect of starch concentration was within the limit of statistical significance, with a *p* slightly higher than 0.01. This result, allied to the low zeta potential value, indicates that emulsions were not stabilized primarily by electrostatic repulsion, and that the predominating stabilizing mechanism was probably steric repulsion.

Regarding particle size, it was possible to observe that, there was no formation of emulsion (emulsion 1) in the absence of starch or Arabic gum. On the other hand, particle size was similar in the presence of these components, regardless of their concentration (Table 2), In other words, none of the variables was statistically significant for these points (Table 3). In addition, particle size was not a function of the concentration of starch or Arabic gum. This indicates that the main factor to determine particle size must have been the preparation methodology, in this case, a high-speed homogenizer at 8,000 rpm for 15 minutes.

For viscosity of emulsions at a shear rate of 1000 1/s (Table 3), three variables proved to be statistically significant (oil, starch and Arabic gum concentrations), since an increase in the amount of any of these components results in an increase in viscosity of the mucilage. Similarly, Mirhosseini et al. [9] also observed that an increase in orange oil, Arabic gum and xanthan gum concentration resulted in an increase in viscosity.

Starch and Arabic gum had a positive effect on η mucilage/ η oil ratio, since the increase in concentration of these components increased viscosity of the mucilage. It bears reminding that, although one of the central points presented a value very different from the others, resulting in a high error (as previously explained), this point did not compromise the analysis of the data of the fractional experimental design. Statistical analysis with or without the inclusion of this point presented the same significant variables, starch and Arabic gum.

The same variables were significant for both responses for statistical analyses of the amount of oil released and stability of each emulsion after 15 days, as expected (Table 3). The variables that presented statistically significant effect were starch and Arabic gum concentrations. In the case of % oil released, the effect of both variables was negative, because the larger the concentration of starch and of Arabic gum, the more stable the emulsion. Unlike other effects within the limit of statistical significance (90% confidence level, i.e., a *p*-value close to 0.01), such as the effect of starch concentration on ζ (*p* = 0.125) and effect of oil concentration on particle size (*p* = 0.120), the effects of starch and Arabic gum concentrations on oil released response were considered statistically significant, even with a *p*-value slightly higher than 0.01 (*p* = 0.108). As described in Materials and Methods, this approach was chosen due to the larger measurements errors associated to visual analyses, avoiding exclusion of variables that, in a more conservative statistical analysis, would not present a significant effect and would not be selected in a screening design of experiments.

After analyzing the results of the first experimental design, we decided to perform a second experimental design, this time a full-factorial design, employing the starch and Arabic gum concentrations as independent variables, which were the only significant variables for stability of emulsions after 15 days and for amount of released oil. As lemon oil and DSS concentrations did not present a statistically significant effect on stability of emulsions and on some of their important properties (Δρ,ζ, particle size, η mucilage/ η oil ratio), this means that they can be fixed at some value, within the tested range; which is more convenient from the point of view of the process evaluated.

### Full-factorial experimental design

This full sequential experimental design set the oil/water ratio at 50% in volume. The high fraction of oil allows reduction of total volume of emulsion, which is an advantage to the beverage industry, implying lower transportation costs. Based on results of the first experimental design, as DSS was not a significant variable for emulsion stability, it was not used this time, which would also represent a reduction in production costs in a large-scale plant. In their studies, Mirhosseini et al. [9] and Tesch et al. [7] obtained stable emulsions for beverages in the absence of surfactant, showing that the option of removing surfactant from the composition is corroborated by other works in literature.

Finally, we opted for varying the concentration of starch and Arabic gum from 0% to 20% in mass of starch or Arabic gum by mass of water, since it was observed in the previous design that viscosity in emulsion with higher concentration (up to 30%) was very high. This is one of the advantages of the strategy of sequential design of experiments. Variables and their conditions are tested with a screening strategy in a two-level fractional factorial experimental design. This is followed by a full-factorial design; allowing adjustment of variable levels according to responses obtained in the first plan, and also enable investigation of interactions between variables that presented some influence [13].

The same responses in the fractional factorial experimental design were analyzed: difference between density of mucilages and density of oil phase with SAIB (Δρ),ratio of emulsion surface tension and mucilage surface tension (γ emulsion/ γ mucilage), droplets ζ, particle size, viscosity of emulsions measured at a shear rate of 1000 1/s, ratio of viscosity of mucilages and viscosity of the oil blend (η mucilage/ η oil), amount of oil released (% oil released) and stability after 15 days of production of the emulsions. The results obtained from the two-level full-factorial experimental design for both variables evaluated (concentration of starch and Arabic gum) are presented in Table 4.

**Table 4:** Responses obtained in the two-level full-factorial experimental design to evaluate the effects of starch and Arabic gum concentrations.

Emulsion	Starch(x_1_)	Arabic Gum(x_2_)	Δρ (muc-oil)(g/cm^3^)	γ emulsion(mN/m)	γ mucilage(mN/m)	γ emulsion/ γ mucilage	ζ(mV)	Particle size (nm)	Viscocity at γ^.^ = 1000 1/s (cP)	η mucilage/ η oil	Oil released(%)	Stability after 15 days(%)
1	-1	-1	0.08	27.1	47.5	0.571	-53.5	8	15	0.008	25	50
2	+1	-1	0.10	41.0	31.4	1.306	-10.3	2531	124	0.058	4	92
3	-1	+1	0.14	46.5	41.6	1.118	-23.9	2065	129	0.065	0	100
4	+1	+1	0.15	51.0	33.0	1.545	-14.1	2885	516	0.185	3	94
5	0	0	0.14	42.7	33.3	1.282	-10.9	2422	151	0.054	9	81
6	0	0	0.14	42.2	32.9	1.283	-10.6	2796	146	0.053	6	87
7	0	0	0.19	42.4	32.8	1.293	-10.5	2529	134	0.043	10	81
Average at the central points			0.16	42.4	33.0	1.290	-10.7	2582	144	0.050	8	83
Standard deviation at the central points			0.03	0.3	0.3	0.01	0.2	193	9	0.006	2	3
Relative standard deviation at the central points (%)			18.4	0.6	0.8	0.5	2.0	7	6	12	25	4

Triplicate in the central points were used to calculate the average, standard deviation and relative standard deviation.

Again, we were able to observe success in achieving the goal of equaling densities of both phases with addition of SAIB (weighting agent) to the oil phase, due to low values of Δρ (Table 4).

As observed in the previous experimental design, the behavior of unstable emulsions that presented lower surface tension than their respective mucilages (for example, γ mucilage = 47.5 mN/m and γ emulsion = 27.1 mN/m, for emulsion 1) (Table 4) was observed again and is attributed to release of oil from droplets to the emulsion surface. Therefore, the γ emulsion/ γ mucilage ratio is an important response to indicate stability of the system and to characterize it.

The measure of the ζ (Table 4) was useful as a characterization of the magnitude of electrostatic effects in the system, but not a good indicator of emulsion stability, as observed in the fractional factorial experimental design. Again, despite the high value of ζ for emulsion with no starch nor Arabic gum (emulsion 1), it was very unstable. Similar to this study, the research of Tesch et al. [7] observed that electrostatic repulsion did not govern stabilization of emulsions prepared with modified starch. In this case, the main mechanism for stabilization through these components (modified starches) is likely steric repulsion.

We observed the same result of the previous experimental design regarding particle size. Droplets had an average size between 2 μm and 3 μm, again with exception for the first emulsion, which was too unstable (Table 4). Again, emulsions presented a narrow particle size distribution, which benefits emulsion stability as explained before [4].

While analyzing viscosity of emulsions at a shear rate of 1000 1/s (Table 4), we observed that, as in the previous experimental design, all emulsions were pseudoplastic (viscosity decreased with the increase of shear rate), differently from mucilages and oil blend, which are Newtonian fluids (the viscosity remained constant with the increase in the shear rate).

Regarding the η mucilage/ η oil ratio (Table 4), we observed that, for both experimental designs, a viscosity ratio around 0.050 favors emulsion stability. In addition to this ratio, viscosity of both phases (oil and mucilage) is important to confer stability to the emulsion, as previously discussed. Note that viscosity of the oil blend in this second set of experiments was higher than in the first, due to the different lot of SAIB used.

The variables “amount of oil released” and “stability after 15 days” presented opposite behaviors, since the release of oil due to droplets coalescence implies emulsion destabilization. Thus, all conditions leading to increase of emulsion stability necessarily lead to decrease of oil released by the emulsion.

Analyses of the effects of independent variables (starch and Arabic gum concentration and interaction between them) on the previously specified responses are presented in Table 5. As one can observe, starch and Arabic gum concentrations are statistically significant to the γ emulsion/ γ mucilage ratio, in that increase in their concentration results in an increase in surface tension ratio, due to their role as stabilizer of these two products.

**Table 5:** Effect (± standard error) of the independent variables on the ratio between the surface tension of the emulsion and of the mucilage (γ emulsion/ γ mucilage), ζ, particle size, viscosity, ratio between the viscosity of the mucilage and of the oil+SAIB (η mucilage/ η oil), amount of oil released and stability of the emulsion after 15 days, in the full-factorial experimental design.

Factor	γ emulsion/ γ mucilage	ζ(mV)	Particle size(nm)	Viscosity at γ^.^ = 1000 1/s (cP)	η mucilage/η oil	Oil released(%)	Stability after15 days (%)
Mean/Interc.	**1.200 ± 0.043**	**-19.1 ± 4.2**	**2177 ± 211**	**174 ± 15**	**0.067 ± 0.008**	**8 ± 1**	**84 ± 1**
Starch	**0.581 ± 0.114**	26.5 ± 11.2	1672 ± 559	**248 ± 40**	**0.085 ± 0.022**	**-9 ± 2**	**18 ± 3**
Arabic gum	**0.393 ± 0.114**	12.9 ± 11.2	1206 ± 559	**253 ± 40**	**0.092 ± 0.022**	**-13 ± 2**	**26 ± 3**
Starch x Arabic gum	-0.154 ± 0.114	-16.7 ± 11.2	-852 ± 559	**139 ± 40**	0.035 ± 0.022	**12 ± 2**	**-24 ± 3**

Statistically significant variables (*p*-value < 0.05) are highlighted in bold.

Statistical analysis of the variables (using 95% confidence level) on ζ is also described in Table 5. Again, we observed the same behavior of the fractional experimental design, indicating that the ζ value was unable to translate into stability, which is more affected by steric hindrance due to relatively high size of both modified starch and gum.

In the absence of either starch or Arabic gum, there was no emulsion formation (experiment 1), as already noticed in the fractional experimental design. On the other hand, particle size was similar in the presence of these variables, regardless of their concentration. (Table 4). The effect of starch is within the confidence limit of 95% (*p* = 0.058), and that of Arabic gum is quite close to the confidence limit of 90% (*p* = 0.120). In other words, with a less rigorous statistical analysis, these two variables become significant, which explains the need for these components to form the emulsion. Probably, the main factor to determine particle size was emulsion preparation methodology, in this case, a high-speed homogenizer at 8,000 rpm for 15 minutes, as occurred in the first design.

Statistical analysis of viscosity of the emulsions at a shear rate of 1000 1/s (Table 5) shows that both starch and Arabic gum concentration, as well as the interaction between them, presented a statistically significant positive influence, as an increase in the amount of any of the components results in an increase in viscosity of the system. As for the η mucilage/ η oil ratio (Table 5), the starch and the Arabic gum are statistically significant variables, but not the interaction between them. Once again, an increase in concentration of starch and Arabic gum, which act as thickeners and stabilizers, increases the η mucilage/ η oil ratio, since increase in concentration of any of these components results in an increase in viscosity of the mucilage.

Finally, we analyzed significant variables for the amount of released oil and the stability of the emulsion after 15 days (Table 5), to discover which response could be a better indicator of emulsion stability. The two variables, concentration of starch and Arabic gum, as well as the interaction between them, were statistically significant for both analyses. In their studies, Mirhosseini et al. [9] observed that a higher amount of Arabic gum resulted in a decrease in turbidity loss rate, which, in turn, was related to emulsion stability. These results were similar to the ones obtained in our study, in which a higher amount of starch and Arabic gum resulted in an increase in stability, here translated as the % oil released, and stability after 15 days.

**Fig 1:**
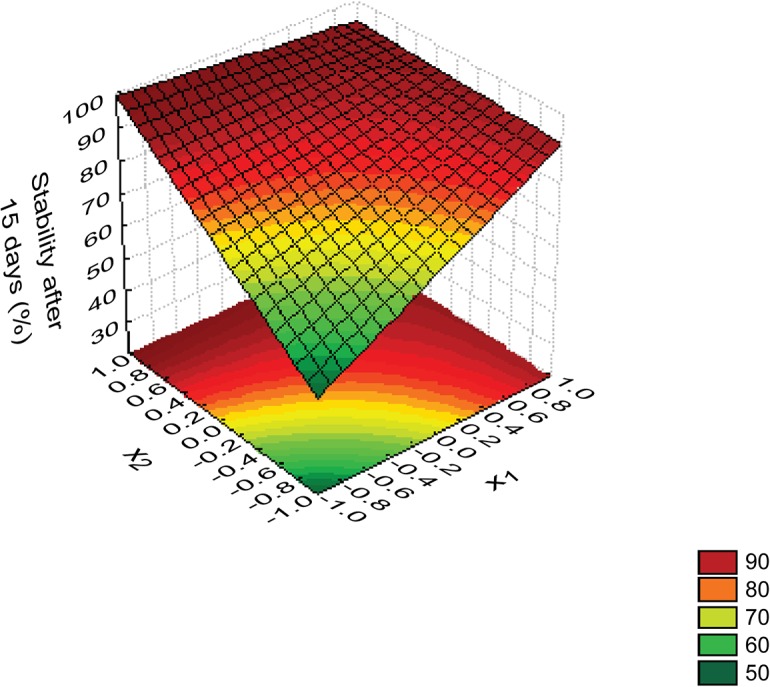
Response surface obtained from the model for stability after 15 days (%) (= *83*.*6* + *9*.*0* x_1_ + *13*.*0* x_2_ - *12,0* x_1_ x_*2*_, where x_1_ and x_2_ are the normalized values of starch and Arabic gum concentrations).

We could obtain a linear mathematical model with a high adjustment quality for each of these new responses, indicated by the correlation coefficient (*R* ^2^) values and validated by ANOVA. The response surface obtained for stability after 15 days is presented in Fig. 1. Through response surface analysis, we could verify that stable emulsions with a stability of around 90% could be obtained both for point 4 of the experimental design, with starch and Arabic gum at the level (+1) and for points 2 and 3, containing only starch or only Arabic gum (points at which one of them is in the level +1 level and the other is in the level −1), respectively. This shows that there is no need to use two stabilizers to improve emulsion stability. Since stability is related to the amount of oil released, both emulsions with starch and Arabic gum and those with only one of these components had a smaller amount of oil released.

The *R* ^2^ of the stability model was around 0.98, which means that the model could explain 98% of total variation in the experimental data. The model was also validated by ANOVA. The value of *F*_calculated_ for the model was 61.29, higher than the tabulated value: *F*_DF Reg, DF Res, α_ = *F*_3, 3, 0.05%_ = 9.28, where DF is the degree of freedom for the regression (DF_Reg_) and for the residues (DF_Res_), respectively (with a probability of 95% or α = 0.05). In other words, a confidence level of 95%, a *F*_calculated_ higher than the *F*_tabulated_ indicates that the model is significant [13]. The model for stability after 15 days was also validated through comparison between experimental data and predicted data (Table 6). Relative errors were low for all experiments and were adjusted without showing a trend, confirming that the model was good enough to represent the experimental data.

**Table 6:** Experimental data and predicted by the model for stability after 15 days (%), for the viscosity of the emulsions at a shear rate of 1000 1/s and for the emulsion/mucilage surface tension ratio.

Emulsion	Starch(x_1_)	Arabic gum(x_2_)	Stability after 15 days (%)(Experimental)	Stability after 15 days (%)(Model)	Relative error(%)	Viscosity at γ^.^ = 1000 1/s (cP)(Experimental)	Viscosity at γ^.^ = 1000 1/s (cP)(Model)	Relative error (%)	γ emulsion/ γ mucilage(Experimental)	γ emulsion/ γ mucilage(Model)	Relative error(%)
1	-1	-1	50	49.6	0.9	15	-7.4	149.5	0.571	0.712	-24.7
2	+1	-1	92	91.6	0.5	124	101.6	18.1	1.306	1.294	0.9
3	-1	+1	100	99.6	0.4	129	106.6	17.4	1.118	1.106	1.1
4	+1	+1	94	93.6	0.5	516	493.6	4.3	1.545	1.688	-9.3
5	0	0	81	83.6	-3.2	151	173.6	-14.9	1.282	1.200	6.4
6	0	0	87	83.6	3.9	146	173.6	-18.9	1.283	1.200	6.5
7	0	0	81	83.6	-3.2	134	173.6	-29.5	1.293	1.200	7.2

Relative error (%) = ((experimental data2014predicted by model) / experimental data) x 100.

**Fig 2:**
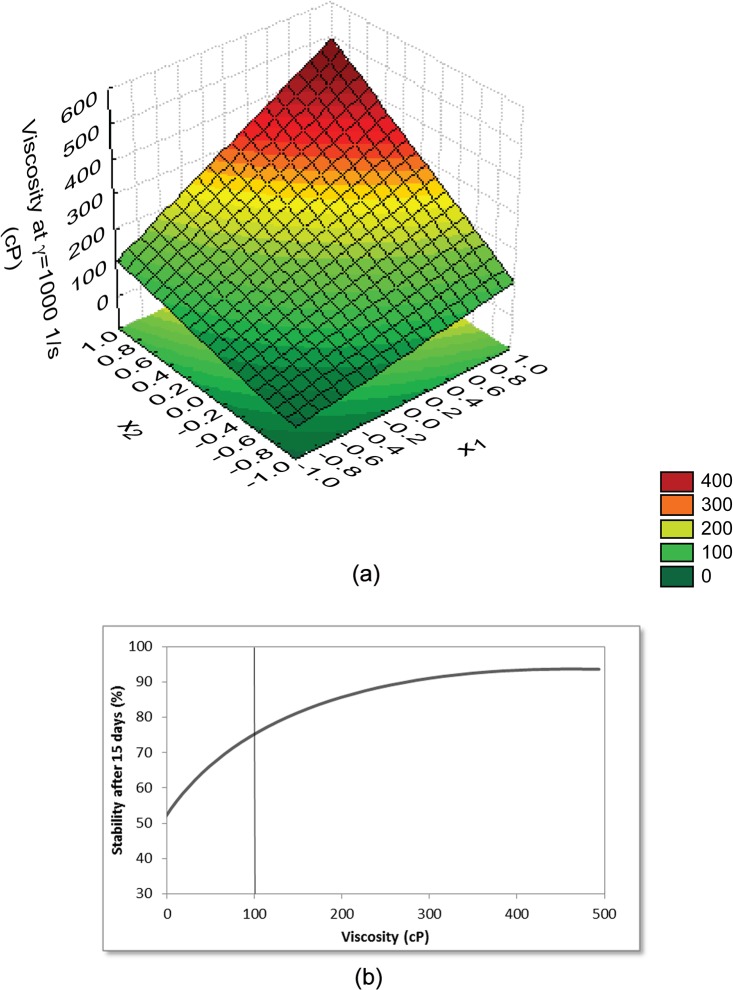
Results for viscosity of the emulsions at a shear rate of 1000 1/s (cP). (a) Response surface obtained from the model for cP (= *173*.*6* + *124*.*0* x_1_ + *126*.*5* x_2_ + *69*.*5* x_1_ x_2_, where x_1_ and x_2_ are the normalized values of starch and Arabic gum concentrations). (b) Viscosity curve versus stability after 15 days.

Comparison of different variables was conducted, as one of the main goals of this work was to discover which variable would allow prediction of stability. We observed that the only response for which starch and Arabic gum concentration and interaction between them were significant, both for stability after 15 days and amount of oil released, was viscosity of the emulsions at a shear rate of 1000 1/s. Viscosity constituted a linear model with *R* ^2^ above 0.97 and validated by ANOVA. The *R* ^2^ equal to 0.97 means that the model could explain 97% of the total variations in the experimental viscosity data. The response surface obtained for the viscosity model at a shear rate of 1000 1/s is presented in Fig. 2(A). These models show that viscosity is not correlated to emulsion stability throughout the evaluated range; yet is suggestive of stability. In other words, there is a minimum viscosity value of around 100 cP, below which emulsion will be very unstable (stability inferior to ca. 75%), as shown in Fig. 2(B).

The model was also validated by ANOVA. The value of *F*_calculated_ = 29.88 was higher than the tabulated value: *F*_DF Reg, DF Res, α_ = *F*_3, 3, 0.05%_ = 9.28. Relative errors between the experimental data and those predicted by the model for viscosity are presented in Table 6. With exception of the first point, all others errors were around 20%, including points 2, 3 and 4, which represent more stable emulsions. The error of the first point can be explained by the much lower viscosity of this emulsion, which makes it difficult for the model to adjust such a large dispersion of data, from 15 cP to 500 cP.

**Fig 3:**
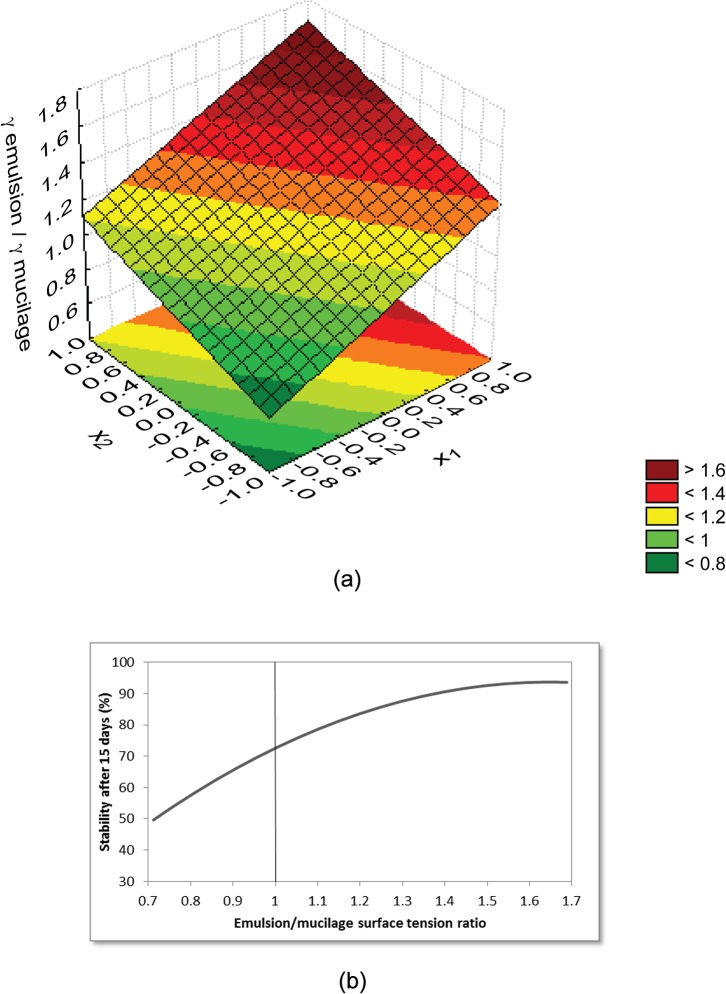
Results for emulsion/mucilage surface tension ratio. (a) Response surface obtained from the model for γ emulsion/ γ mucilage (= *1*.*200* + *0*.*291* x_1_ + *0*.*197* x_2_, where x_1_ and x_2_ are the normalized values of starch and Arabic gum concentrations). (b) Curve of the emulsion/mucilage surface tension ratio versus stability after 15 days.

In addition to viscosity, the γ emulsion/ γ mucilage ratio also showed interesting behavior. In all emulsions studied, we noticed that, if this ratio is superior to 1, that is, if the surface tension of the emulsion is higher than the mucilage surface tension, the emulsion is stable, as previously explained. Below 1, the emulsion will be unstable, as can be observed in the model response surface presented in Fig. 3(A). This Figure shows that whenever the ratio is higher than 1, the emulsion will be stable, but it does not mean that, the higher the value, the more stable the emulsion. On the other hand, when the ratio is lower than 1, the emulsion formed will be unstable, as shown in Fig. 3(B).

The γ emulsion/ γ mucilage ratio also produced a linear model with a *R* ^2^ of 0.90 and validated by ANOVA. The model could explain 90% of the total variations in the experimental data. Added to that, the value of *F*_calculated_ for the model was 15.65, higher than the tabulated value: *F*_DF Reg, DF Res, α_ = *F*_2, 4, 0.05%_ = 6.94, indicating that the model can be considered significant. Relative errors between the experimental data and those predicted by the model are presented in Table 6. There was a high degree of concordance between values measured and predicted. Except for the first point (a very unstable emulsion), which presented a higher error, the relatively low errors of the other points confirmed that the model was good enough to represent experimental data. This discrepancy of the first point is because, without the interaction between starch and Arabic gum, which was not significant (Table 5), the first point was not as well adjusted, as it would be if it had presented the interaction. Considering the interaction, the relative error between the values measured and predicted for the ratio γ emulsion/ γ mucilage ratio would be −11.3%.

## Conclusions

We obtained emulsions with stability superior to 15 days containing only one stabilizer agent and without surfactant (emulsions 2 and 3), which present a direct impact in terms of reduction of process costs, process simplification and raw material availability. It is also possible to reduce stabilizer concentration to 16 g of starch or Arabic gum/ 100 g water. In addition, we observed that addition of a surfactant agent is not required.

The zeta potential of the droplets was not related to emulsion stability, indicating that the stabilizing mechanism was mainly steric hindrance due to the presence of a layer of starch or gum at the droplets’ surface. All emulsions were pseudoplastic and it we discovered that a viscosity over 100 cP is needed to increase stability. In addition, surface tension of the emulsion should be higher than the surface tension of the respective mucilage. Both answers were selected to represent the behavior of emulsions in terms of stability and could be used as tools for an initial screening of more promising formulations.

Using modified starch or Arabic gum, we were able to obtain concentrated emulsions (50% of dispersed oil) with stability superior to 15 days, presenting only 5% release of the oil phase. This concentration is much higher than that used by the beverage industry, which currently employs an oil amount between 15% and 20%.
